# Five Fraction Image-Guided Radiosurgery for Primary and Recurrent Meningiomas

**DOI:** 10.3389/fonc.2013.00213

**Published:** 2013-08-20

**Authors:** Eric Karl Oermann, Rahul Bhandari, Viola J. Chen, Gabriel Lebec, Marie Gurka, Siyuan Lei, Leonard Chen, Simeng Suy, Norio Azumi, Frank Berkowitz, Christopher Kalhorn, Kevin McGrail, Brian Timothy Collins, Walter C. Jean, Sean P. Collins

**Affiliations:** ^1^Department of Neurosurgery, Georgetown University Hospital, Washington, DC, USA; ^2^Department of Radiation Medicine, Georgetown University Hospital, Washington, DC, USA; ^3^Department of Pathology, Georgetown University Hospital, Washington, DC, USA; ^4^Department of Radiology, Georgetown University Hospital, Washington, DC, USA

**Keywords:** radiosurgery, meningioma, toxicity, fractionation, treatment outcome

## Abstract

**Purpose:** Benign tumors that arise from the meninges can be difficult to treat due to their potentially large size and proximity to critical structures such as cranial nerves and sinuses. Single fraction radiosurgery may increase the risk of symptomatic peritumoral edema. In this study, we report our results on the efficacy and safety of five fraction image-guided radiosurgery for benign meningiomas.

**Materials/Methods:** Clinical and radiographic data from 38 patients treated with five fraction radiosurgery were reviewed retrospectively. Mean tumor volume was 3.83 mm^3^ (range, 1.08–20.79 mm^3^). Radiation was delivered using the CyberKnife, a frameless robotic image-guided radiosurgery system with a median total dose of 25 Gy (range, 25–35 Gy).

**Results:** The median follow-up was 20 months. Acute toxicity was minimal with eight patients (21%) requiring a short course of steroids for headache at the end of treatment. Pre-treatment neurological symptoms were present in 24 patients (63.2%). Post treatment, neurological symptoms resolved completely in 14 patients (58.3%), and were persistent in eight patients (33.3%). There were no local failures, 24 tumors remained stable (64%) and 14 regressed (36%). Pre-treatment peritumoral edema was observed in five patients (13.2%). Post-treatment asymptomatic peritumoral edema developed in five additional patients (13.2%). On multivariate analysis, pre-treatment peritumoral edema and location adjacent to a large vein were significant risk factors for radiographic post-treatment edema (*p* = 0.001 and *p* = 0.026 respectively).

**Conclusion:** These results suggest that five fraction image-guided radiosurgery is well tolerated with a response rate for neurologic symptoms that is similar to other standard treatment options. Rates of peritumoral edema and new cranial nerve deficits following five fraction radiosurgery were low. Longer follow-up is required to validate the safety and long-term effectiveness of this treatment approach.

## Background

Meningiomas are commonly benign tumors with a generally favorable prognosis (1). However, without treatment they may progress locally, compressing adjacent structures and causing neurologic deficits. They pose a unique clinical challenge due to their large size and variable anatomical locations within the skull (1). Surgical resection of the entire tumor, when possible without neurologic injury, is the standard of care with a 10-year local control of 80% or higher (2–9). For subtotally resected or recurrent tumors, conventionally fractionated radiation therapy (1.8–2.0 Gy per fraction) to approximately 54 Gy improves local control (2, 4, 6–8).

More recent experience suggests a role for single fraction stereotactic radiosurgery (SRS) (12–18 Gy) as a primary treatment for well selected, small meningiomas or as adjuvant treatment for residual disease (10–12). In cases where single fraction SRS has been appropriately utilized, results have been excellent, demonstrating equivalent local control to both conventional radiation therapy and surgical resection for select groups of meningioma patients (10, 11). Patients with large tumors (>7.5 cc) have a poor prognosis with this approach, and unacceptably high rates of local failure (10, 11).

Single fraction radiosurgery, however, may increase the risk of symptomatic peritumoral edema and/or cranial nerve injury (10, 12, 13). This risk of peritumoral edema may be increased in tumors that are large, recurrent, adjacent to large veins, and/or basally located (10, 13–19). Conventional fractionated radiation therapy has been employed to treat these patients. The gross tumor volume (GTV) is typically targeted with a margin of 2–5 mm to adjust for set-up inaccuracy. Due to these large planned treatment volumes (PTVs), treatment is generally fractionated over 25–30 sessions to limit toxicity to adjacent normal structures. Due to the long natural history of this disease, it is essential to maximize post-treatment quality of life by preventing treatment related adverse outcomes while minimizing neurological symptoms associated with tumor progression. It is possible that some of the adverse effects of single fraction radiosurgery for large tumors may be mitigated by limited fractionation.

The CyberKnife is an image-guided, frameless, SRS platform. The frameless configuration allows for staged treatment, and it has been successfully utilized to treat a wide variety of intracranial tumors including meningiomas (8, 9, 20). In this retrospective study, we report our preliminary results with five fraction image-guided radiosurgery as a treatment for meningiomas, either as monotherapy or as an adjuvant to surgical resection. This treatment was conducted with the belief that its accurate and highly conformal delivery would minimize peritumoral edema and cranial nerve toxicity.

## Materials and Methods

### Patient selection and treatment

**Figure 1:**
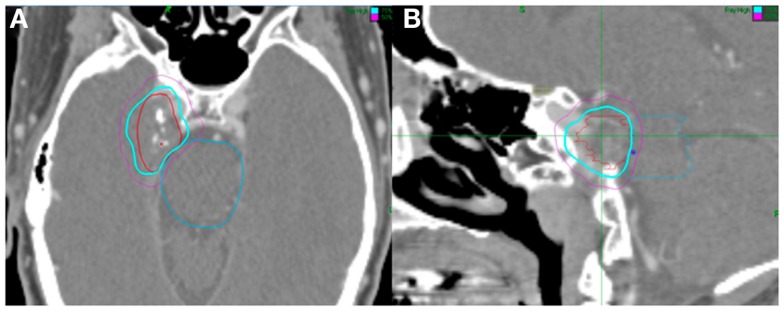
**Fifty-three-year-old man with a right Meckel cave meningioma**. He presented with right facial pain. The decision was to proceed with radiosurgery. Treatment planning axial **(A)** and sagittal **(B)** computed tomography images demonstrating the GTV (red), brainstem (blue), and chiasm (yellow). Isodose lines shown as follows: blue 79% (prescription) and purple 50%. Note proximity of the meningioma to the brainstem. The tumor was treated with 2500 cGy in five fractions and his pain resolved.

We performed a retrospective review of patients with benign meningiomas treated with CyberKnife SRS from December 1st, 2007 to February 1st, 2011 by SPC and BTC. Patients who had undergone SRS for intracranial meningiomas with or without surgical resection were included in the present study. Patients with atypical or malignant meningiomas were excluded from this study. All patients were treated by an interdisciplinary team of radiation oncologists and neurosurgeons. High resolution CT images were obtained from all patients for pre-treatment planning with target volumes, and critical structures were manually delineated by the treating neurosurgeon (Figure 1). The treating isodose and prescription dose were determined by the treating radiation oncologist in consultation with the treating neurosurgeon, and took into account the target volume, proximity to critical structures, and previous treatment history. In most cases, the dose was prescribed to the isodose surface that encompassed the margin of the tumor. Treatment plans were generated using an inverse planning method by the CyberKnife treatment software (Multiplan, Accuray).

### Outcomes assessment

Patients were tracked as part of routine clinical follow-up by the interdisciplinary team. MRI scans were obtained at pre-defined intervals, every 6 months for the first year, and then yearly thereafter, unless acute changes in neurological status warranted immediate imaging. Neurological symptoms were clinically assessed and recorded by the treating neurosurgeons. Peritumoral edema was assessed on T2 weighted and FLAIR MRI sequences. Patient steroid requirements were assessed at each clinical follow-up visit.

### Statistical analysis

All statistical analyses were performed utilizing SPSS Statistics v19 (IBM). Statistical analysis was performed in order to identify pre-treatment and treatment variables that correlated with post-treatment peritumoral edema. Due to the relatively small sample size, Fisher’s Exact Test was used for categorical variables, while Spearman’s Rho was employed for examining the interaction between continuous variables and post-treatment peritumoral edema. For analysis of volume and dose, due to the small sample sizes, patients were stratified as being over or under the median and a Chi-square test was employed. Alpha was set to 0.05 to yield a 95% confidence interval (CI) for all statistical tests.

## Results

### Patient and treatment characteristics

Thirty-eight patients were identified as having undergone treatment for intracranial meningiomas and were subsequently included in the current study (Table 1). Twenty-nine (79%) of the patients were female and nine (24%) were male. The median age at time of treatment with radiosurgery was 64 years. Thirteen (34%) patients had undergone prior surgery, of which five were classified as gross total resection and eight were classified as subtotal resections. The remaining 24 patients had received no previous surgical or non-surgical interventions and were treated without pathologic confirmation. Twenty-seven (71%) of the tumors were primary, while 11 (29%) were recurrent. The tumors occurred at a variety of intracranial sites (Table 2), with an almost even number of basal and non-basal tumors, 22 (58%) and 16 (42%) respectively. The median tumor volume was 3.83 mm^3^ (range, 1.08–20.79 mm^3^). The median isodose was 82% (70–90%) which was treated with a median prescription dose of 2500 cGy (2500–3500 cGy) and resulted in a median percent tumor coverage of 99.5% (Table 3).

**Table 1:** **A summary of patient characteristics for patients included in the study**.

Characteristic	*N* = 38	(%)
Race/ethnicity
Caucasian	24	(63)
African American	11	(29)
Hispanic	1	(3)
Asian	2	(5)
Gender
Female	29	(76)
Male	9	(24)
Age at radiosurgery
Mean	62	
Median	64	
Extent of resection
Gross total	5	(13)
Subtotal	8	(21)
No surgery	24	(63)

**Table 2:** **A summary of tumor characteristics for all tumors included within the study**.

Characteristic	*N* = 38	(%)
Primary vs. recurrent
Primary	27	(71)
Recurrent	11	(29)
Location: general
Basal	22	(58)
Non-basal	16	(42)
Location: specific
Bifrontal	1	(3)
Cavernous sinus	7	(18)
Cerebellopontine angle	5	(13)
Falcine	2	(5)
Falcotentorial	1	(3)
Lateral ventricle	1	(3)
Meckel’s cave	2	(5)
Middle cranial fossa	1	(3)
Parafalcine	2	(5)
Parasagittal	5	(13)
Parietal convexity	1	(3)
Parietal lobe	1	(3)
Petroclival	2	(5)
Posterior fossa	1	(3)
Sphenoid wing	2	(5)
Suprasellar	1	(3)
Temporal lobe	3	(8)
Volume (cc)
Min	1.08	
Max	20.79	
Mean	6.22	
Median	3.84	

**Table 3:** **A summary of treatment characteristics for patients treated on a frameless stereotactic radiosurgical system**.

Characteristic	*N* = 38	Characteristic	*N* = 38
Rx dose (cGy)		Percent tumor covered
Min	2500	Min	97.4
Max	3500	Max	99.9
Mean	2691	Mean	99.3
Median	2500	Median	99.5
Isodose line (%)		Non-zero beams
Min	70	Min	88
Max	90	Max	259
Mean	82	Mean	175
Median	82	Median	174
Homogeneity index		Collimator (mm)
Min	1	Min	5
Max	1.39	Max	15
Mean	1.22	Mean	11
Median	1.2	Median	10
New conformality index		
Min	1.32		
Max	2.25		
Mean	1.66		
Median	1.61		

### Complications and neurological symptoms after SRS

Acute toxicity after SRS treatment included symptoms such as headaches, fatigue, and nausea. Headaches were the most common complication with nine patients (23.7%) complaining of headaches at the end of treatment. Four patients (10.5%) experienced fatigue, and only one patient (2.6%) complained of nausea. Twenty-four patients (63.2%) presented with neurological symptoms prior to therapy (Table 4). These neurological symptoms included facial pain, hearing loss, diplopia, proptosis, vertigo, facial numbness, and reduced visual acuity. After SRS, neurological examination revealed complete resolution of neurological symptoms in 14 patients (58.3%), continued symptoms in eight patients (33.3%), and recurrence of symptoms after initial improvement in two patients (8.3%). Only one patient (2.6%) developed a new deficit, facial numbness, immediately after radiation, which resolved after a few days. Otherwise, no new neurological deficits were observed after SRS.

**Table 4:** **A summary of changes in neurological deficits**.

Deficit	Pre-SRS	Improvedpost-SRS	Recurrence after initialimprovement	Continued Sx post-SRS
Facial pain	9	5	2	2
Hearing loss	1	0	0	1
Diplopia	4	4	0	0
Proptosis	2	0	0	2
Vertigo	2	2	0	0
Facial numbness	4	3	0	1
Reduced visual acuity	2	0	0	2

*All neurological deficits were noted by the treatment team on either clinical exam or through direct questioning of the patient*.

Facial pain was the most common presenting neurological symptom pre-SRS treatment. Of the nine patients (37.5%) who presented with facial pain, five patients (55.6%) were asymptomatic after radiation, two patients (22.2%) had continued symptoms, and another two patients (22.2%) had recurrent facial pain after initial improvement. Diplopia, vertigo, and facial numbness improved in the majority of patients. Proptosis and reduced visual acuity did not improve with treatment.

### Local control rate and peritumoral edema

Twenty-four patients (63.2%) who underwent SRS showed no change in tumor size, while 14 patients (36.8%) showed a decrease in tumor size resulting in a crude radiographic local control rate of 100% of the meningiomas treated with SRS (Table 5).

**Table 5:** **(A) A comprehensive table detailing individual patient outcomes with regards to pre-treatment therapies, radiation dosage, and subsequent clinical outcomes. (B) A summary of individual patient factors and whether patients had pre-treatment or post-treatment peritumoral edema**.

**(A)**						
Patient	Location treated	Surgery	Cumulativedose	Local outcome	Acute toxicity	Post-radiationsteroids
1	Temporal lobe	None	3000	Decreased	Headache	Yes
2	Tentorial	None	3500	Decreased	No	No
3	Posterior Temporal lobe	Subtotal	3000	Stable	No	No
4	Cavernous sinus	Subtotal	2500	Decreased	No	No
5	CPA	None	2750	Stable	No	No
6	CPA	None	2750	Stable	No	No
7	Cavernous sinus	None	2500	Stable	No	No
8	Cavernous sinus	None	2500	Stable	Headache	Yes
9	Parasagittal	Gross total	2500	Stable	Fatigue	No
10	Parietal falcine	Subtotal	2500	Stable	No	No
11	Parietal Parasagittal	Gross total	2500	Stable	Headache	No
12	Petroclival	Subtotal	2500	Stable	Fatigue	No
13	Medial sphenoid wing	Subtotal	2500	Stable	Fatigue and headache	No
14	Middle cranial fossa	None	3000	Stable	Headache	Yes
15	Petroclival	None	2500	Stable	Headache	Yes
16	Cavernous sinus	Subtotal	2500	Decreased	No	No
17	Frontal parafalcine	None	2500	Decreased	No	No
18	Sphenoid wing	None	2500	Decreased	No	No
19	CPA	None	2500	Stable	No	No
20	Parietal convexity	None	2500	Stable	No	No
21	CPA	None	2500	Stable	No	Yes
22	Anterior parafalcine	None	3000	Decreased	Headache and nausea	Yes
23	Bifrontal	None	3000	Stable	No	No
24	CPA	None	3000	Decreased	Headache	No
25	Anterior falcine	Gross total	3000	Decreased	No	No
26	Cavernous sinus	None	2500	Decreased	No	No
27	Falcotentorial	Subtotal	2500	Stable	No	No
28	Posterior fossa	Subtotal	3000	Stable	No	No
29	Posterior Parasagittal	Gross total	2500	Stable	No	No
30	Cavernous sinus	None	2500	Stable	No	No
31	Parafalcine	None	2500	Decreased	No	No
32	Anterior temporal	Gross total	3000	Decreased	Headache	Yes
33	Lateral ventricle	None	3000	Stable	No	No
34	Suprasellar	None	2500	Stable	Fatigue	Yes
35	Cavernous sinus	None	2500	Decreased	Hypesthesia	No
36	Meckel’s cave	None	2500	Stable	No	No
37	Meckel’s cave	None	2750	Decreased	No	No
38	Parietal lobe	Gross total	3000	Stable	No	No

**(B)**						
**Patient**	**Anatomicalclassification**	**Volume (cc)**	**Recurrence**	**Adjacentto vein**	**Pre-treatmentperitumoral edema**	**Post-treatmentperitumoral edema**

1	Non-basal	1.08	No	No	No	No
2	Non-basal	1.6	No	Yes	No	Yes
3	Non-basal	16.7	Yes	No	Yes	Yes
4	Basal	5.56	Yes	Yes	Yes	Yes
5	Basal	1.37	No	No	No	No
6	Basal	2.56	No	Yes	No	No
7	Basal	4.05	No	No	No	No
8	Basal	12.19	No	No	No	No
9	Non-basal	11.24	Yes	No	Yes	Yes
10	Non-basal	6.48	Yes	No	No	No
11	Non-basal	6.44	Yes	Yes	No	No
12	Basal	2.12	Yes	No	No	No
13	Basal	20.17	No	No	No	No
14	Basal	2.14	No	Yes	No	Yes
15	Basal	20.79	No	No	No	No
16	Basal	13.82	No	Yes	Yes	Yes
17	Non-basal	6.43	No	No	No	No
18	Basal	5.48	No	No	No	No
19	Basal	10.84	No	No	No	No
20	Non-basal	3.24	No	No	No	No
21	Basal	12.13	No	No	No	Yes
22	Non-basal	1.17	No	No	No	No
23	Non-basal	6.59	No	No	No	No
24	Basal	1.53	No	No	No	No
25	Non-basal	3.59	Yes	Yes	Yes	Yes
26	Basal	13.07	No	No	No	No
27	Non-basal	4.68	No	No	No	No
28	Basal	11.83	Yes	No	No	No
29	Non-basal	2.63	Yes	No	No	Yes
30	Basal	2.65	No	No	No	No
31	Non-basal	2.611	No	No	No	Yes
32	Non-basal	3.04	Yes	Yes	No	No
33	Non-basal	3.628	No	Yes	No	No
34	Basal	2.62	No	No	No	No
35	Basal	1.38	No	Yes	No	No
36	Basal	4.97	No	No	No	No
37	Basal	1.90	No	Yes	No	No
38	Non-basal	1.89	Yes	Yes	No	No

Intracranial edema is commonly managed with oral steroids, and oral steroid requirements were measured as a surrogate for post-radiation peritumoral edema. Symptomatic, acute, post-radiation edema requiring steroids occurred in six patients (15.8%). In addition, two patients (5.3%) were given steroids due to evidence of post-radiation edema on MRI, but without any clinical signs of toxicity (Table 5).

Pre-SRS radiographic peritumoral edema continued to be observed in five patients (13.2%) on follow-up MRI imaging. Of these patients, four (10.5%) had recurrent tumors following a subtotal or gross resection, and three (7.9%) had a radiological tumor volume greater than 10.0 cc (Table 5). A total of 10 patients had post-treatment radiographic peritumoral edema, with new onset being observed in five patients (13.2%). On univariate statistical analysis, only pre-treatment peritumoral edema (*p* = 0.001) and adjacency to a large vein (*p* = 0.045) correlated with post-treatment peritumoral edema (Table 6).

**Table 6:** **A statistical analysis of variables associated with peritumoral edema**.

Pre-treatment characteristic	Likelihood ratio	*p*-Value
Pre-treatment peritumoral edema	15.77	0.001
Anatomical classification	1.28	0.293
Adjacent to vein	4.83	0.045
Volume (cc)	0	1
Recurrence	2.77	0.116
Cumulative dose	0.002	0.968

*p-Values are for two-sided Fisher’s Exact Test*.

## Discussion

Our results show that fractionated SRS may provide similar local control with minimal toxicity and excellent quality of life. Headaches, fatigue, and nausea were the only three acute complaints, all of which resolved over time. Headaches were the most common complication, present in 23.7% of our patients, which is consistent with other studies (12). Nausea was the least common, present in only one patient. This trend has also been observed in previous studies (21, 22).

In this study several patients presented with neurological symptoms and the majority responded to treatment with minimal toxicity at 2 years of follow-up. The present response rate of neurological symptoms compares favorably to similar studies with Gamma Knife (17, 21). Kondziolka et al. noted that five patients in their series of 99 cases had new or worsened deficits occurring 3–31 months after radiosurgery, while Chang et al. reported two cases out of 140 experiencing worsened deficits. Most tellingly, Kondziolka et al. reported that 67 out of 70 patients reported that their treatments were subjectively “successful” on an outcomes questionnaire, indicative of a high preservation of quality of life post-SRS (21). Uniquely, we have found an excellent response of tumor-associated facial pain to five fraction radiosurgery. While documented in other studies involving single fraction radiosurgery, our results suggest that a five fraction approach can also yield a beneficial reduction in tumor-associated trigeminal neuralgia (23–25). Other studies have suggested that recurrence of these symptoms typically occurs within 2 years, and is more likely to recur for malignant skull base tumors, with the mechanism of relief being decompression of affected nerve roots (24, 25).

Stereotactic radiosurgery was well tolerated with few post-treatment complications. As previously mentioned, other studies have suggested a relationship between tumor volume and post-SRS edema and complications (26). However, we found no correlation found between tumor volume, margin dose, and the presence of complications, which is similar to findings in other studies (12, 14, 22). Furthermore, it may be that if such a relationship does exist between large tumor volume and complications, that it may be mitigated in part through dose fractionation like in the present study.

At roughly 2 years, none of the patients developed local failures, and 14 showed a decrease in tumor size that may be correlated favorably with local control, although this has not been conclusively shown (27). There is a high degree of variability in volume reduction post-radiosurgery with studies reporting rates less than 20% and over 60%, ultimately the implications and the time course of post-radiosurgery volume reduction need to be further studied to ascertain its prognostic implications (21, 28). With regards to local control, control rates for meningiomas post-radiosurgery typically require longer follow-up for thorough assessment, with many studies placing the 10-year rate of local control at 84% (11, 22, 29).

Only 13% of the patients developed new onset post-SRS peritumoral edema, with 26% of patients developing it overall. In addition, only 2.6% of the patient group receiving five fraction radiosurgery had symptomatic peritumoral edema. These results are in agreement with other papers on the use of hypofractionated radiosurgery for meningiomas, and compares favorably to an average of 5–10% of patients developing symptomatic edema in other studies (12, 21, 30, 31). In one such study by Kollova et al. edema was more common in tumor volumes greater than 10 cm^3^ (26). However the present study and others have suggested that simple tumor volume is not a significant contributor to post-radiation peritumoral edema, which may be in fact more due to the interface between meningioma and cortical tissue rather than gross volume (21, 32).

## Conclusion

Stereotactic radiosurgery is a safe and effective treatment for benign intracranial meningiomas with or without surgical resection. Dose fractionation is well tolerated, and may offer equivalent local control to single session SRS. Fractionation may offer particular benefit to patients with large tumors located in critical locations or in other high-risk patients. Further studies are warranted to fully ascertain the potential benefits and risks of dose fractionation for SRS therapy of meningiomas, and its ultimate impact on local control.

## Conflict of Interest Statement

Brian Timothy Collins and Sean P. Collins have received honoraria from Accuray Inc. The other co-authors declare that the research was conducted in the absence of any commercial or financial relationships that could be construed as a potential conflict of interest.
